# In memoriam: Massimo Santini

**DOI:** 10.1093/europace/euaf028

**Published:** 2025-02-26

**Authors:** Giuseppe Boriani

**Affiliations:** Cardiology Division, Department of Biomedical Metabolic and Neural Sciences, University of Modena and Reggio Emilia, Via del Pozzo 71, 41124 Modena, Italy

**Figure:**
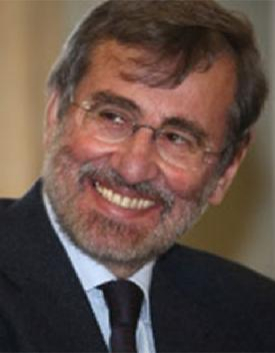


Massimo Santini, MD, a distinguished cardiologist and a pivotal figure in the field of arrhythmias and cardiac pacing, passed away on 9 January 2025, at the age of 79. Born in Rome, he dedicated his entire career to advancing medical science and improving patient care in his beloved city. He graduated in Medicine from the University ‘La Sapienza’ in Rome and completed his specialization in Cardiology at the University of Perugia.

Dr Santini began his career as a cardiologist focusing on arrhythmias and cardiac pacing at San Camillo Hospital in Rome. He later served as Chief of Cardiology at S. Filippo Neri Hospital, where he led a team of cardiologists across all domains of clinical cardiology, with a particular focus on cardiac arrhythmias and pacing techniques.

Massimo Santini held numerous prestigious positions at both national and international levels, including the presidency of the World Society of Arrhythmias, the Italian Association of Cardiac Pacing and Arrhythmias (AIAC), and the Italian Association of Hospital Cardiologists (ANMCO).

He was a pioneer in the technological evolution of cardiac pacing, defibrillation, and resynchronization therapy, playing a leading role in advancing innovations in electrical therapies for arrhythmias, remote monitoring, and cardiac resynchronization. His contributions to international research groups significantly shaped the field over the last 50 years.

His enthusiasm for clinical research was infectious, and he was deeply committed to mentoring young electrophysiologists, inspiring them to establish research teams focused on optimizing electrical therapies in clinical practice. He was instrumental in the leadership of one such group, named ‘Clinical Service’, which united numerous research centres across Italy. Their collaborative efforts enhanced the clinical evaluation of pacing, defibrillation, and resynchronization therapy in terms of outcome research, as well as improved knowledge on the intricate relationship between atrial fibrillation, left ventricular dysfunction, and thromboembolic risk in patients with cardiac implantable electronic devices.^1–4^ I had the privilege of being part of this research group, and I fondly remember Massimo as an inspiring and visionary leader. He encouraged fresh ideas, fostering an environment where younger colleagues felt empowered to contribute to research initiatives. In meetings, he balanced a relaxed and approachable demeanour with intellectual rigor, ensuring that discussions were productive and inclusive. Whenever disagreements arose, Massimo's natural sense of humour and vast clinical experience helped navigate the team towards consensus.

Massimo Santini was among the first to support, together with Renato Ricci, a wide implementation of remote monitoring for improving both quality of life and outcomes of patients with cardiac implantable devices.^5,6^ He strongly promoted at the international level the adoption of remote monitoring of cardiac implantable electronic devices as a standard of care, coupling the efficiency of device clinics with patients’ needs to ensure the highest levels of safety.^7–9^

Since 1984, he organized the biennial ‘Progress in Clinical Pacing’ congress in Rome, a landmark event that spanned 18 editions and brought together cardiologists from around the world to discuss the latest advancements in pacing, arrhythmias, syncope, and heart failure therapies. For three decades, this congress was a highly anticipated event, held in the enchanting setting of Rome in early December.

Massimo Santini's professional and clinical activities were deeply intertwined with Rome, where he cared for patients from all walks of life, from the most humble individuals to prominent politicians. When speaking with colleagues, he often recounted patient stories, blending clinical insights with profound humanity. His ability to communicate with patients and empower them to understand their conditions was a defining trait of his career.

He was also the founder of the non-profit organization ‘The Heart of Rome’ (‘Il Cuore di Roma’), aimed at educating the public about cardiovascular disease risks and the importance of prevention and evidence-based treatments. This initiative led to significant scientific contributions, such as a large-scale screening study that collected electrocardiogram (ECG) data from over 24 000 adolescents in Rome, providing unique insights into the correlations between ECG abnormalities and anthropometric parameters in this population.^10^ Under the auspices of the same organization, Massimo Santini co-authored a remarkable book ‘My Electric Heart’ (‘Il Mio Cuore Elettrico’) written together with his nephew, Luca Santini, an active electrophysiologist at G.B. Grassi Hospital in Rome. This freely available online book serves as a unique patient-friendly guide, explaining arrhythmias and their treatments, including pacemakers, defibrillators, and ablation therapies, in a clear and accessible manner.

Massimo Santini will be remembered for his charismatic presence, unwavering dedication, and the relentless passion he brought to his clinical and scientific endeavours. His legacy earned him the affectionate title of ‘The Emperor of Rome’, a testament to his leadership, empathy, and profound impact on everyone he encountered throughout his remarkable life.

## Data Availability

No data available in relationship with this article.
